# Acute-on-chronic liver failure: a global disease

**DOI:** 10.1136/gutjnl-2020-323973

**Published:** 2021-02-25

**Authors:** Martin Schulz, Jonel Trebicka

**Affiliations:** 1 Translational Hepatology, Department of Internal Medicine I, Goethe University Frankfurt, Frankfurt am Main, Germany; 2 European Foundation for the Study of Chronic liver Failure, EFCLIF, Barcelona, Spain

**Keywords:** cirrhosis, liver failure

Acute-on-chronic liver failure (ACLF) is a frequent complication in hospitalised patients with liver cirrhosis. A large body of data has been published in recent years, demonstrating that acute decompensation constitutes a dramatic turning point in the course of cirrhosis, with development of ACLF being the most severe form of acute decompensation (AD).^1^ Within the last decades, heterogeneous definitions of ACLF have been proposed in different regions of the world, that is, the European European Association for the Study of the Liver - Chronic Liver Failure (EASL-CLIF) definition, the NASCELD definition in North American and the East Asian APASL criteria. Due to those, epidemiological data on ACLF are heterogenous and not easy to compare.

**Figure 1:**
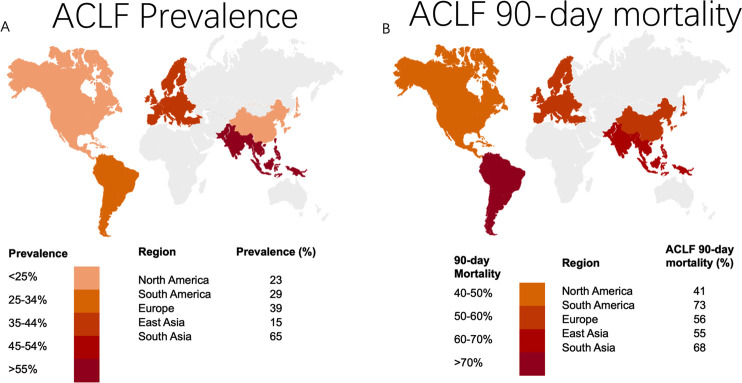
The figure depicts the known world-wide prevalence (A) and 90-day mortality (B) of acute-on-chronic liver failure (ACLF) reviewed in the meta-analysis Mezzano *et al.* 3

In *Gut*, Mezzano and colleagues have undertaken huge efforts to homogenise and compare the existing evidence.^2^ They present an extensive systematic review and meta-analysis on the burden of ACLF worldwide (figure 1A), which constitutes the largest epidemiological study on this subject to date.^3^ The authors were able to identify 30 prospective and retrospective cohort studies from around the world, which include 43 206 ACLF patients and 140 835 patients without ACLF. Strengths of this study are its scale and the robustness of data, which highlight the global significance of ACLF for patients and healthcare systems.

The authors chose the EASL-CLIF ACLF criteria as the more balanced between east and west. This meta-analysis demonstrated, that 35% of patients admitted with decompensated cirrhosis worldwide presented an ACLF at hospital admission, with a 60% mortality in the first 90 days. Interestingly, the 90-day mortality rates differed by region, showing the highest mortality in South America (73%) and South Asia (68%), rendering this study the first to map geographic differences in ACLF outcomes (figure 1B). As in the CANONIC study, kidney failure seems to be the most common with almost 50% worldwide, whereas respiratory failure was the least common organ failure reported with 11%. However, this may be under-represented due to the portion of patients followed by hepatologists, while intensive care physicians work less frequently in this field. Interestingly, alcohol was the most frequent aetiology of underlying cirrhosis with 45% worldwide, showing the highest prevalence in Europe. Importantly, this should again raise the awareness of the community to dedicate efforts and funds to this stigmatised and neglected population of patients. Regional differences were also reported in the prevalence of alcohol consumption as the precipitating event triggering ACLF with the highest in East Asia and North America with 30%, followed by Europe with 25%. Yet, the most frequent ACLF trigger events portrayed in this meta-analysis were bacterial infections in 35%, followed by gastrointestinal bleeding 22% and alcohol 19% globally. In Europe and South Asia, almost 50% of ACLF patients showed bacterial infection as ACLF precipitating event. This finding was also confirmed in a recent multicentric prospective study, Predicting Acute on Chronic Liver Failure (PREDICT).^4 5^ The large prospective PREDICT trial, which has not been included in this meta-analysis since its results have only been published recently, has classified and evaluated precipitating events prospectively and their role on outcomes.^5^ PREDICT included 1273 European patients, who were non-electively hospitalised with acute decompensation. It showed bacterial infection and severe alcoholic hepatitis, either alone or in combination, accounted for almost all (96%–97%) acute decompensations and ACLF in its cohort.^5^ Furthermore, it was able to show that the type of ACLF trigger did not influence patient’s outcome, whereas number of precipitating events did. Yet, objective criteria for the classification of precipitants in the studies included in this meta-analysis were missing. Therefore, it has to be stated, that the data are probably not entirely consistent with the PREDICT study and possibly reality.

Nevertheless, this systematic review and meta-analysis draws the attention to the global significance of ACLF. Recent concepts are, that ACLF is one form of acutely decompensated cirrhosis, but PREDICT could characterise the other forms.^4^ A very severe form is pre-ACLF, mainly driven by systemic inflammation, which has since been validated in Chinese cohorts.^6^ A different phenotype in AD is unstable decompensated cirrhosis, which is mainly driven by portal hypertension.^7 8^ In addition, stable decompensated cirrhosis patients constitute a major part of patients acute decompensation. These different courses of disease are not reflected in studies included in this meta-analysis. It will be a major challenge for investigations in the future to allow early identification of acute decompensation phenotypes and to stratify patients for individual risk for disease progression. One step in this direction was the introduction of the M10LS20 algorithm, which allows bed-side stratification of patients with advanced chronic liver disease based on Model for Endstage Liver Disease (MELD) and liver shear-wave elastography (L-SWE).^9^

In summary, this meta-analysis, conducted by Mezzano and colleagues, is the first study to gather worldwide epidemiological data on prevalence and mortality of ACLF to systematically evaluate the global burden of disease (see figure 1). Even though the authors chose to restrict study inclusion by EASL-CLIF definition of ACLF, the significance of the general conclusions drawn from this meta-analysis remain most relevant. ACLF is highly prevalent worldwide in hospitalised patients with acute decompensation and is associated with high short-term mortality. This fact urges for unified international criteria defining acute decompensation and recommendations on patient’s management.
